# Amino acid substitutions affecting aspartic acid 605 and valine 606 decrease the interaction strength between the influenza virus RNA polymerase PB2 '627' domain and the viral nucleoprotein

**DOI:** 10.1371/journal.pone.0191226

**Published:** 2018-01-16

**Authors:** Ho-Pan Hsia, Yin-Hua Yang, Wun-Chung Szeto, Benjamin E. Nilsson, Chun-Yeung Lo, Andy Ka-Leung Ng, Ervin Fodor, Pang-Chui Shaw

**Affiliations:** 1 Centre for Protein Science and Crystallography, School of Life Sciences, The Chinese University of Hong Kong, Shatin, N.T., Hong Kong, China; 2 Sir William Dunn School of Pathology, University of Oxford, Oxford, United Kingdom; Universidad Autonoma de Madrid Centro de Biologia Molecular Severo Ochoa, SPAIN

## Abstract

The influenza virus RNA genome is transcribed and replicated in the context of the viral ribonucleoprotein (vRNP) complex by the viral RNA polymerase. The nucleoprotein (NP) is the structural component of the vRNP providing a scaffold for the viral RNA. In the vRNP as well as during transcription and replication the viral polymerase interacts with NP but it is unclear which parts of the polymerase and NP mediate these interactions. Previously the C-terminal ‘627’ domain (amino acids 538–693) of PB2 was shown to interact with NP. Here we report that a fragment encompassing amino acids 146–185 of NP is sufficient to mediate this interaction. Using NMR chemical shift perturbation assays we show that amino acid region 601 to 607 of the PB2 ‘627’ domain interacts with this fragment of NP. Substitutions of these PB2 amino acids resulted in diminished RNP activity and surface plasmon resonance assays showed that amino acids D605 was essential for the interaction with NP and V606 may also play a partial role in the interaction. Collectively these results reveal a possible interaction surface between NP and the PB2 subunit of the RNA polymerase complex.

## Introduction

Influenza is a contagious respiratory disease that causes recurring annual epidemics and occasional pandemics. According to the World Health Organization, influenza epidemics claim the life of 250,000 to 500,000 people every year. Influenza A virus genome comprises of eight segments of viral RNA (vRNA) encoding 10 major proteins and several auxiliary polypeptides [1,2]. Each of the eight RNA segments of the virus is packaged into a viral ribonucleoprotein (vRNP) complex consisting of multiple nucleoproteins (NPs) and one heterotrimeric RNA polymerase, which binds the partially complementary 5’ and 3’ termini of the vRNA. Transcription and replication of the viral RNA genome is carried out by the viral polymerase in the context of the vRNP complex [3,4].

The RNA polymerase of influenza A virus consists of three subunits: PA, PB1 and PB2. The PB1 subunit is at the centre of the polymerase complex and contains the motifs characteristic of RNA-dependent RNA polymerases. The PA subunit contains an N-terminal endonuclease and a large C-terminal domain, connected through a 70 amino acid long linker. The PB2 subunit consists of multiple domains, including the cap-binding, mid-link, ‘627’ and nuclear localization signal (NLS) domains in the C-terminal two thirds of the molecule. The PA endonuclease and PB2 cap-binding domains are involved in the cap-snatching process by binding to and cleaving, respectively, host 5’ capped RNAs, producing capped RNA fragments used as primers by the polymerase for the initiation of transcription of viral genes. The PB2 ‘627’ domain (amino acids 538–693) is named after the host range determinant amino acid 627, which is almost invariably a glutamate in avian but a lysine in mammalian-adapted influenza A viruses [5]. The function of the PB2 ‘627’ domain in the context of the polymerase complex is still unclear, although it was shown recently that a polymerase mutant lacking the ‘627’ domain can mediate core polymerase functions in vitro but cannot replicate viral RNA in cells [6].

NP is the most abundant protein in the RNP complex. Its primary function is to provide a scaffold for vRNA and the complementary RNA (cRNA) replicative intermediate to facilitate the formation of the double helical RNPs [7–9]. NP has been reported to interact with all three polymerase subunits [10–13] and has been suggested to act as a switching factor from transcription early in infection to genome replication late in infection [14–16]. However, more recent research suggested that NP is an elongation factor rather than a regulator of transcription or replication [17].

The sites of interaction between NP and PB2 are poorly mapped. Three independent regions of NP (amino acids 1–161, 255–340, 340–465) were found to bind PB2, while the C-terminus of NP (amino acids 465–498) inhibits the interaction [18]. On the other hand, two fragments of PB2 (amino acids 1–269 and 580–683) were shown to interact with NP [12]. However, these sites of interaction were mapped by deletion mutagenesis and cover a significant portion of both proteins (75% of NP and 50% of PB2). Several studies reported reduced interaction between NP and PB2 of avian influenza viruses in mammalian cells that can be restored by the E627K adaptive mutation [13,19,20]. This led to the hypothesis that PB2 amino acid 627 is involved in NP-PB2 interactions. However, another report suggested that the apparent stronger binding of NP with PB2 observed in co-immunoprecipitation with a PB2 E627K mutant is a consequence of increased polymerase activity instead of higher binding affinity between NP and PB2 [21]. In addition, PB2 627E was found to restrict polymerase activity in mammalian cells in an NP-independent manner suggesting that reduced binding between avian PB2 and NP in mammalian cells is not the primary reason for the low activity of avian influenza virus polymerase in mammalian cells [22]. To gain a better insight into the interplay between NP and PB2 here we investigate the interaction between NP and the PB2 ‘627’ domain further and identify amino acids 605 and 606 of PB2 that is involved in an interaction with amino acid region of 146–185 of NP.

## Results

### Amino acids 601–607 of the PB2 ‘627’ domain are involved in interaction with NP

**Fig 1:**
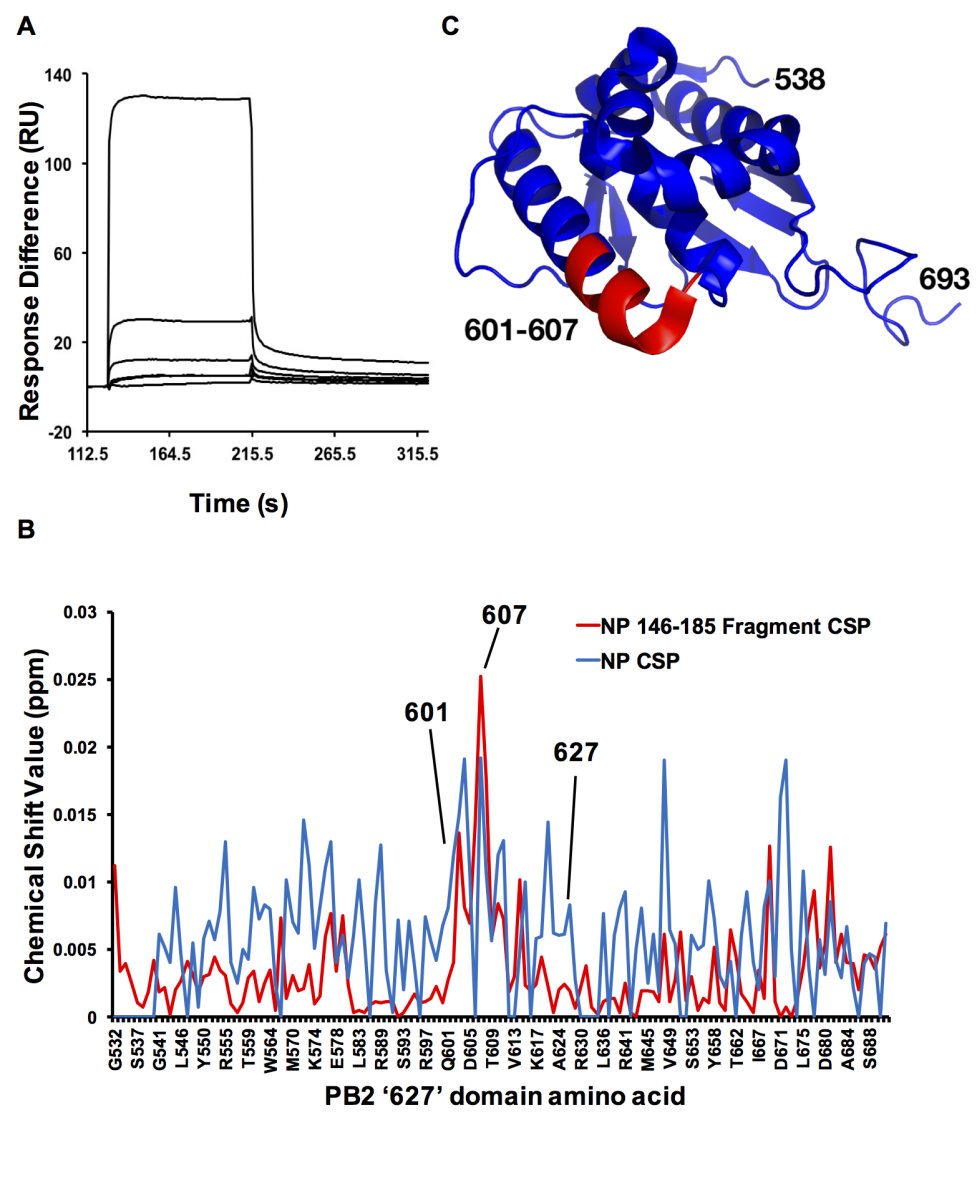
Interaction between NP fragment 146–185 and PB2 involves PB2 amino acids 601 to 607. (A) SPR sensorgrams of interaction between NP fragment 146–185 and PB2 ‘627’ domain. ‘627’ domain was immobilized on a CM5 sensor chip and NP 146–185 of 0, 312.5, 625, 1250, 2500, 10000 nM was injected. (B) ^15^N-labelled PB2 ‘627’ domain was perturbed by either full-length NP (blue, colour code: #0000FF) or NP fragment 146–185 (red, colour code: #FF0000), and the chemical shift of each amino acid was recorded. For some amino acids on the PB2 ‘627’ domain the perturbation data were unavailable and the values were assigned as zero. (C) Amino acids 601 to 607 (highlighted in red, colour code: #FF0000) of the PB2 ‘627’ domain are located on an alpha-helix adjacent to the characteristic phi-loop of the domain (PDB ID: 2VY7).

Previously, our group reported that NP interacts with the PB2 ‘627’ domain and identified NP R150 to be involved in the interaction, mutation of this results in an abolished interaction [20]. To address the question whether a fragment of NP containing R150 is sufficient for the interaction, we expressed and purified NP fragment 146–185 with a His-SUMO tag and PB2 ‘627’ domain with His tag in a bacterial expression system using established protocols [5]. We tested the binding of these two proteins in SPR experiments with BIAcore 3000. The purified PB2 ‘627’ domain was immobilized on a CM5 sensor chip and purified NP fragment 146–185 was injected into the system at increasing concentrations. The kinetic parameters were measured, the binding curves were fitted and the affinity constant was calculated. The curves fitted best with 1:1 Langmuir model. The NP peptide bound to the PB2 ‘627’ domain with an affinity of 927 nM (k_a_: 7.23×10^4^ M^-1^s^-1^, k_d_: 0.067 s^-1^) (Fig 1A). To identify putative amino acids in PB2 involved in the interaction with NP, we expressed ^15^N-lablled PB2 ‘627’ domain in bacterial expression system and performed a chemical shift perturbation (CSP) experiment, using either full-length NP or NP fragment 146–185 as ligand. The resultant perturbation patterns by both the peptide and full-length NP were comparable, amino acids 532–537 shown in Fig 1B were the remain of the His-tag after cleavage. In particular, PB2 amino acids 601–607 were highly perturbed in both cases. Therefore, these seven amino acids were selected for further experiments. These amino acids (Fig 1C, highlighted in red) were located on an alpha helix adjacent to the characteristic phi-loop of the PB2 ‘627’ domain. Perturbed amino acids downstream of 607 were excluded from further experiments because they are buried in the crystal structure of RNA polymerase (PDB ID: 4WSB) while amino acids 601–607 are exposed and readily accessible.

These results show that NP region 146–185 plays an important role in mediating NP-PB2 ‘627’ domain interactions but suggest that other regions of NP also contribute to the binding. Furthermore, these results are consistent with previous findings that the NP-PB2 ‘627’ domain interaction is direct without involving RNA [20].

### Substitutions of amino acids 601–607 of the PB2 ‘627’ domain disrupts RNP activity

**Fig 2:**
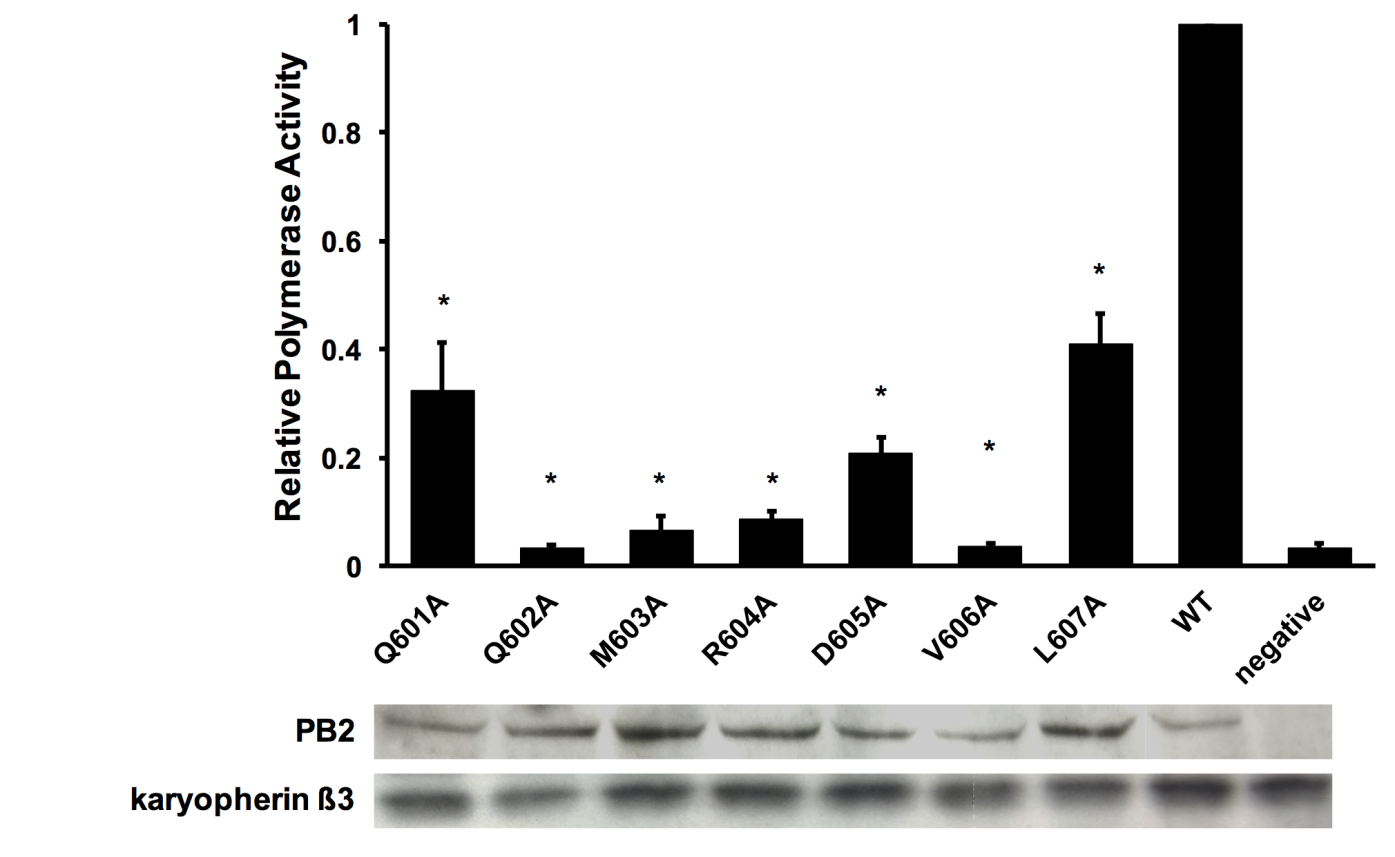
Substitutions of amino acids 601 to 607 of the PB2 ‘627’ domain disrupt RNP activity. Plasmids expressing wild-type or mutant PB2, PA, PB1, NP and a vRNA encoding luciferase were co-transfected along with a plasmid expressing GFP as control. The overall polymerase activity is represented by a ratio of luminescence signal to GFP signal. The activity of polymerase with wild-type PB2 was set to 1. Level of PB2 expression of each mutant was detected by anti-PB2 polyclonal antibodies in Western blotting with karyopherin ß3 as loading control. The bar represents the mean ratio ± standard deviations from three independent experiments. *The p-values are smaller or equal to 0.05 in two-tailed Student’s *t*-test.

Next, we addressed the role of amino acids 601–607 in PB2 by measuring the RNP activity with PB2 mutants in a luciferase assay [23]. The RNP activity was represented as a ratio of luminescence to GFP level, and the activity of RNP with wild-type PB2 was set as 1. Transfection of wild-type PB2 resulted in detectable luminescence, indicating that the RNP was functional (Fig 2). All the alanine mutants had dramatically reduced RNP activity. We conclude that amino acids 601 to 607 of PB2 are important for RNP activity.

### PB2 amino acids 605 and 606 are involved in viral transcription and replication in an NP dependent manner

**Fig 3:**
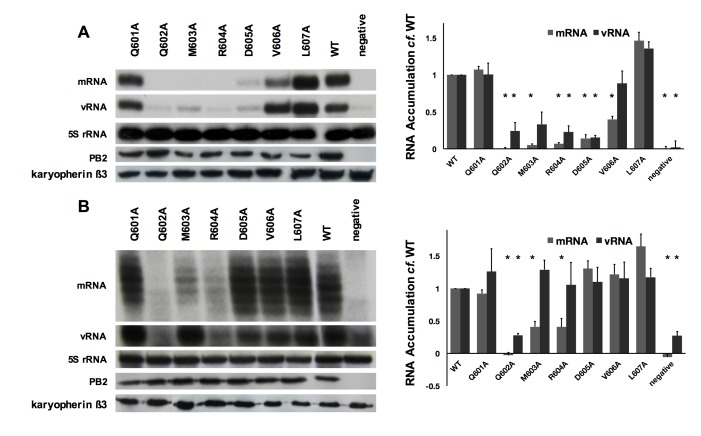
Substitutions of amino acids 605 and 606 in PB2 affect RNP activity in an NP-dependent manner. (A) Primer extension analysis of vRNA and mRNA form vRNP reconstitution assays using a full-length segment 6 vRNA template. PB2 was omitted as negative control and the levels of 5S rRNA acted as internal control. Level of PB2 expression of each mutant was detected by anti-PB2 polyclonal antibodies in Western blotting with karyopherin ß3 as loading control. Quantification was performed by phosphoimage analysis of data from three independent experiments. The mRNA and vRNA levels of WT were set to 1. (B) Similar to (A) but without NP and using a truncated 47 nt-long vRNA-like template. *The p-values are smaller or equal to 0.05 in two-tailed Student’s *t*-test.

As reported by a previous study, influenza virus polymerase can replicate and transcribe templates with length shorter than 76 nt in the absence of NP [17]. Therefore, substitutions of polymerase amino acid solely involved in binding NP but otherwise not contributing to transcription and replication should have no effect on short vRNA templates. To test whether PB2 amino acids 601–607 were solely involved in NP-binding, we co-expressed a full-length segment 6 vRNA or its internally truncated 47 nt-long version with wild-type or mutant polymerases. There were three observations for the PB2 variants. First, Q601A and L607A did not show a decrease in RNP activity as in luciferase reporter assay, the reason of such difference shall be investigated in follow-up study. Second, variants Q602A, M603A and R604A showed a significant drop in the short template analysis, this indicated that the RNP activity loss was due to disrupted polymerase rather than NP-PB2 interaction disruption. Third, variants D605A and V606A showed similar vRNA and mRNA levels as the wild-type in primer extension analysis on the short vRNA template (Fig 3). The activity was significantly reduced for variant D605A on both templates. Variant V606A also showed a 50% reduction in the transcription of the full-length template. This suggested that PB2 amino acid 605 and to some extend 606 are involved in the polymerase function through the binding to NP.

### Substitution of amino acid 605 or 606 of the PB2 ‘627’ domain abolishes its interaction with NP

**Fig 4:**
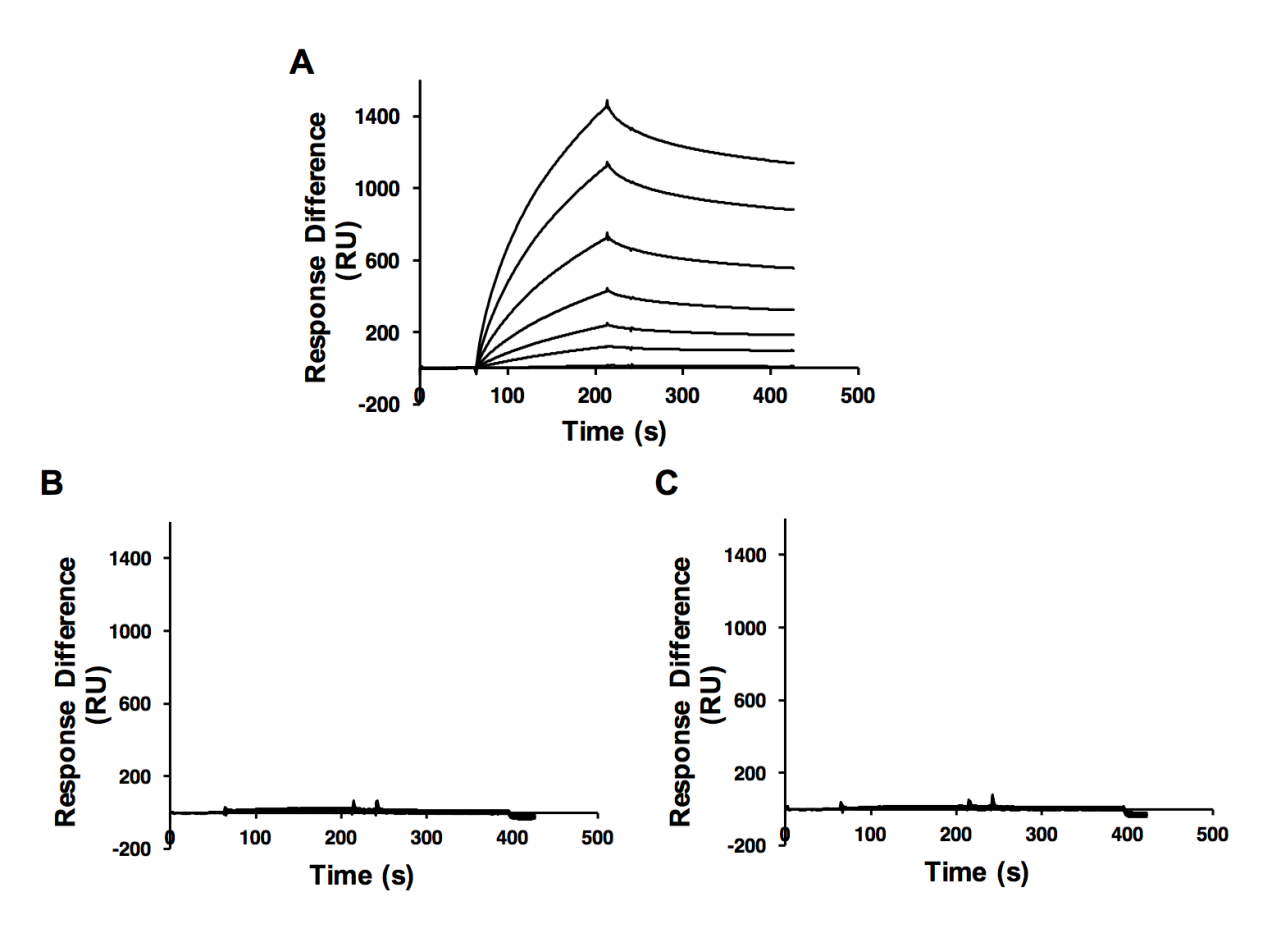
Amino acids 605 and 606 of the PB2 ‘627’ domain are involved in interaction with NP. SPR was performed with wild-type PB2 (A), PB2 variant D605A (B) or PB2 variant V606A (C) using increasing concentrations of NP mutant R416A produced by 2-fold serial dilutions. The NP concentration series covers a range from 7 nM to 8000 nM. Curves produced by 500, 250, 125, 62.5, 31.25, 15.63 and 7.82 nM NP are shown in the sensorgrams.

D605A and V606A variants of the PB2 ‘627’ domain were expressed in a bacterial expression system and purified to homogeneity using established protocols [5]. The purified PB2 ‘627’ mutants were then immobilized onto a CM5 sensor chip by amine coupling. Purified monomeric NP R416A was applied at increasing concentrations, produced by 2-fold serial dilutions, to determine the kinetic parameters of the interaction. The resultant curves were fitted by 1:1 Langmuir model. NP R416A interacted with the wild-type PB2 ‘627’ domain with an affinity of 274 ± 91 nM (Fig 4A), which was similar to the previously reported K_D_ of the interaction between wild-type NP and wild-type PB2 ‘627’ domain (252 nM) [20].

In contrast to the wild-type, the PB2 ‘627’ domain mutants produced flat curves in the binding analysis, even with the highest concentration of NP R416A (Fig 4B and 4C). These data are agreement with the results of the RNP assays and confirm that PB2 amino acids 605 and 606 are involved in the interaction with NP.

**Fig 5:**
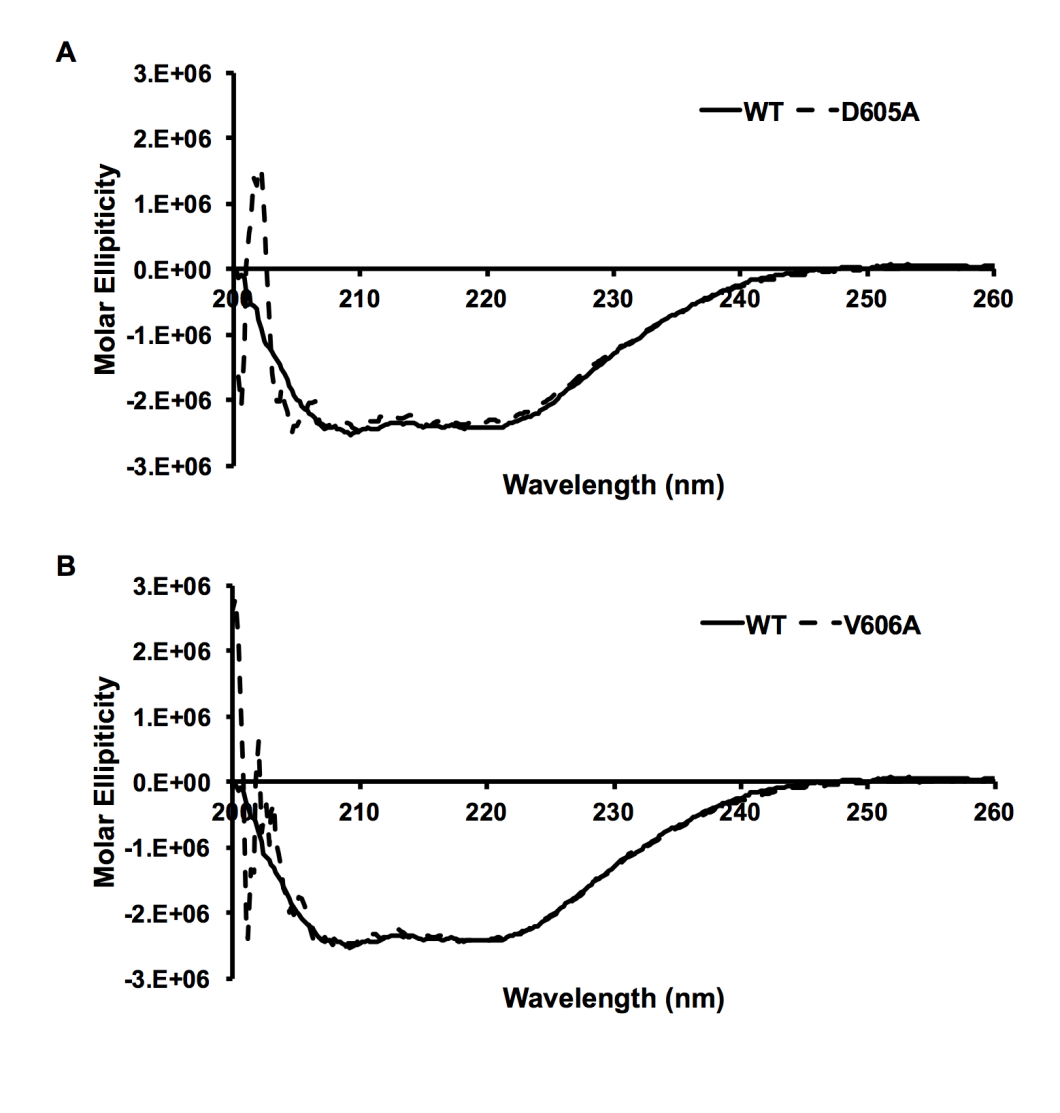
PB2 variants D605A and V606A do not affect the secondary structures of the PB2 ‘627’ domain. Circular dichroism spectroscopy was performed using purified PB2 ‘627’ domain. Spectra were recorded from 260 to 200 nm and were averaged over three measurements. The spectrum obtained for wild-type PB2 is compared with that of PB2 mutant D605A (A) and V606A (B).

To address the possibility that the observed disruption of interaction was due to structural disruption of the domain by the amino acids substitutions, circular dichroism spectroscopy was performed to examine the secondary structures of the mutants. It was found that these two mutants produced similar spectra compared with the wild-type (Fig 5), indicating that the secondary structures of these variants were not significantly disturbed.

## Discussion

In this study we aimed to gain further insight into the interaction of the PB2 polymerase subunit with NP. We show that the PB2 ‘627’ domain interacts with full-length NP as well as an NP fragment encompassing amino acids 146–185. Using NMR chemical shift perturbation experiments we found that PB2 amino acids 601 to 607 were particularly affected by the interaction suggesting the involvement of this region of the PB2 ‘627’ domain in direct interaction with NP. RNP activity assays confirmed the importance of this polymerase region for activity. While substitutions of amino acids 602 to 604 affected activity on both full-length and truncated vRNA templates, substitutions at amino acids 605 and 606 specifically affected activity on the full-length but not the truncated vRNA template that can be transcribed and replicated in the absence of NP. These results suggested that PB2 amino acids 605 and 606 are involved in polymerase function primarily by mediating interaction with NP. Indeed, SPR data support the involvement of these amino acids in interaction with NP.

We have surveyed 19159 influenza A virus PB2 sequences available in the Influenza Research Database. Amino acids 601 to 607 were found to be almost absolutely conserved (identical in more than 19100 sequences analysed), regardless of the host species. This indicates that this region of PB2 is involved in an essential polymerase function which is crucial to the virus. Based on our data we propose that amino acids 605 and 606 of PB2 contribute to polymerase function through mediating interaction with NP. From our previous study, basic amino acid at position 627 or 630 is necessary for the interaction [20]. The low CSP signal of E627 agreed with the previous study, interestingly, we found that PB2 R630 was barely perturbed in NMR chemical shift perturbation experiments by either the full-length NP or the NP 146–185 fragment. Since amino acid R630 is located at a flexible loop in PB2, which may undergo conformational movements in the intermediate exchange regime causing extensive broadening of the NMR signals, we may not observe the HN-N cross peaks of these amino acids in HSQC spectra. As a result, we have not observed the CSP reading of R630 when using full-length NP as ligand. On the other hand, there is a small CSP reading when using an NP 146–185 fragment as ligand, reflecting the change of binding affinity has shifted the interaction to fast exchange regime. The small reading suggested R630 is not involved in binding the proposed region of NP (aa 146–185). These findings are in agreement with the proposal that adaptive mutations at these amino acids affect polymerase-NP interactions indirectly by primarily affecting polymerase activity [22]. The possibility of the involvement of R630 of PB2 in NP-PB2 interaction cannot be excluded but it is possible that aa 601–607 nearby, which showed a much higher CSP signal, may interact with NP and that interaction was proved by SPR (Fig 4).

Although this study did not identify the interacting amino acids on NP, it provides some clues about how the interaction takes place in NP. A fragment of NP comprising amino acids 146–185 exhibited an affinity of 927nM for the PB2 ‘627’ domain where the full-length NP showed an affinity of 252nM for the domain [20]. The affinity of NP fragment for PB2 ‘627’ domain is considerably significant since the fragment only comprises less than 1/10 of the amino acids of NP. These results indicate that NP 146–185 plays an important role in the interaction but other regions also contribute. Previously, we have shown that substitution of R150 in NP to A abolished the RNP activity in an H5 polymerase background, but not an H1 background [20]. This suggested the possible interaction of NP R150 and PB2 D605. Whether two amino acids interact directly and the importance of such interaction requires further study. NP 146–185 is located at the interface of the head and body domains and some of the basic amino acids within this fragment were shown to be important for RNA-binding [24]. Furthermore, in previous studies amino acid S165 was found to be phosphorylated during infection and phosphomimetic mutations were shown to inhibit oligomerisation suggesting that this region of NP is also involved in the regulation of oligomerisation [25]. Further studies are required to understand how this region in NP takes part in RNA binding, oligomerisation and interaction with the PB2 ‘627’ domain.

In our study, both the luciferase reporter assay and RNP reconstitution assay have indicated that substitutions of amino acids D605 and V606 in PB2 resulted in the partial to complete loss of RNP activity. Combining the results from SPR study, the loss of RNP activity is most likely caused by disruption of interaction between NP and PB2 ‘627’ domain. This suggested the interaction is essential for the functioning of the RNP. The recently published atomic structures of the viral RNA polymerase may shed some light on this question [26–28]. It was postulated that the nascent RNA exits the polymerase from a basic channel near the ‘627’ domain [28]. It is possible that the ‘627’ domain interacts with NP to bring NP into close proximity with nascent cRNA and vRNA for encapsidation and vRNP template formation. Therefore, disruption of the interaction between NP and the PB2 ‘627’ domain may thus stop the replication process. The finding that substitutions at PB2 amino acids 605 and 606 specifically interfere with polymerase activity on a full-length vRNA template requiring NP for replication but not on a short template that can be replicated in the absence of NP is consistent with the proposed hypothesis. The next question is how these mutations would affect the interaction between NP and polymerase complex within the host cells. To answer this question, an integrated study of other PB2 regions is required since PB2-NP interaction occurs in multiple sites [12].

To conclude, this work delineates the molecular details of the interaction between NP and the PB2 ‘627’ domain. Amino acids 146–185 of NP were found to interact with the PB2 ‘627’ domain, and this interaction primarily involves amino acids 605 and 606 of PB2 with D605 having more importance in this interaction. The loss of this interaction disrupts RNP activity. Overall our study provides new insights into the association of NP and PB2.

## Materials and methods

### Biological materials

HEK 293 and 293T were obtained from ATCC, Manassas, VA, USA. Cells were maintained in Dulbecco’s Modified Eagle Medium (DMEM) (Life Technologies) supplemented with 10% fetal bovine serum (Life Technologies). The virus strain was A/Hong Kong/156/97. Anti-NP serum was prepared from NP immunized rabbits by Guangdong Medical Laboratory Animal Centre and anti-PB2 and anti-karyopherin ß3 (H-300) serum was purchased from Santa Cruz. pcDNA3a, pcDNA-PA, pcDNA-PB1, pcDNA-PB2 and pcDNA-NP encoding sequences of strain A/Hong Kong/156/97, pPolI-NA and pPolI-NA47 encoding sequences of influenza A/WSN/33 (H1N1) virus have been described previously [22,29]. pPOLI-Luc-RT and pEGFP were the gifts from Professor L.L.M Poon (The University of Hong Kong) and have also been described [23].

### Expression and purification of influenza A virus NP, unlabelled and ^15^N labeled PB2 ‘627’ domain

The sequences encoding ‘627’ domain of PB2 (amino acids 538–693) from A/Hong Kong/156/97 (H5N1) was cloned into pET28a expression vector. The plasmids were transformed into *E*. *coli* BL21(DE3). Protein expression was induced by the addition of 0.4 mM IPTG at 25 ^o^C for 5 h when OD_600_ reached 0.6–0.8. Unlabeled proteins were expressed in LB medium while the ^15^N labeled protein in M9 minimal medium, with the supplement of ^15^NH_4_Cl. The protein was purified by Ni-affinity and gel filtration column chromatography, according to established protocols [5]. Full-length NP of A/Hong Kong/156/97 (H5N1) was expressed and purified as described previously [24].

### Construction and expression of NP fragment 146–185

NP fragment 146–185 was generated by PCR and cloned into pHis-SUMO vector for protein expression. The expression vector is a modified SUMO plasmid with a SUMO protease-recognition site inserted between the SUMO-tag and the protein of interest. The clones were sequenced to ensure that it encodes a His-affinity purification tag, followed by the sequence representing SUMO and NP 146–185. NP fragment 146–185 was expressed in the *E*. *coli* strain BL21 (DE3 soluble) (Novagen). The recombinant protein was purified with a Ni–NTA affinity column (GE Healthcare) in 20mM sodium phosphate (pH 7.0), 200mM NaCl. The SUMO-fusion protein was eluted with increasing concentration of imidazole and the SUMO-tag was removed by SUMO protease digestion overnight at 4°C. Then NP fragment 146–185 was recovered and further purified by a Ni–NTA affinity column and Superdex 75 (GE Healthcare) gel filtration column on the AKTA Fast Protein Liquid Chromatography system (GE Healthcare) in 20mM sodium phosphate (pH 7.0), 200mM NaCl. Protein purity was checked by 15% SDS-PAGE.

### Surface plasmon resonance (SPR) study of the NP-PB2 ‘627’ domain interaction

Purified wild-type or mutant PB2 ‘627’ domain was individually immobilized onto a CM5 sensor chip (GE Healthcare) using amine coupling kit (GE Healthcare) until the response unit (RU) had increased to about 615 from baseline. NP mutant R416A or NP fragment including amino acids 146–185 was injected into the sensor chip at increasing concentrations in 20mM Tris-HCl pH8, 300mM NaCl, 5% glycerol, 0.005% Tween-20. The experiments were performed at 25 ^o^C with BIAcore 3000. Data were analysed with BIAevaluation v 4.1. SPR measurements were fitted with 1:1 Langmuir model to obtain association constants, dissociation constants and affinity of the interaction.

### Backbone resonance assignment and NMR chemical shift perturbation experiments

To identify amino acids with large chemical shift perturbation (CSP), backbone resonance assignment of the PB2 ‘627’ domain in the presence or absence of NP was carried out using a series of triple resonance NMR experiments including the HNCO, HN(CA)CO, HN(CO)CACB, HNCACB, HNCA, HN(CO)CA. All NMR experiments were performed on a Bruker Avance 700 MHz NMR spectrometer with cryoprobe at 298 K. NMR data processing was accomplished using NMRPipe software (version linux9) [30] and analyzed with SPARKY software (version 3.114) [31]. ASSTOOLS software was used to assist the process of backbone assignment. Ambiguous spin assignment table was created by using ASSTOOLS to find the best set of sequentially matching assignments among all ambiguous combinations [32].

The interaction between the PB2 ‘627’ domain and NP was studied using the NMR chemical shift perturbation (CSP) method, in which the interaction was monitored through chemical shift changes in ^15^N-HSQC spectra of ^15^N labeled PB2 ‘627’ domain upon the addition of unlabeled full-length NP or NP fragment 146–185. The full-length NP and NP fragment 146–185 to PB2 ‘627’ domain molar ratios used were 0.5, 0.75, 1, 1.5, 2, and 3. A number of peaks shifted significantly, whereas the positions of the majority of the cross-peaks remained unchanged, indicating that there was a specific interaction between the PB2 ‘627’ domain and NP. The measured chemical shift changes for ^1^H and ^15^N were combined into a composite CSP vector ΔCS [33].

### Influenza polymerase activity analysis by luciferase reporter assay

For transfection 0.125 μg each of pcDNA-PA, pcDNA-PB1, pcDNA-PB2 wild-type or mutant, pcDNA-NP, pPOLI-Luc-RT and pEGFP plasmids were diluted to 12.5 μl in OptiMEM (Life Technologies) and added to 1.05 μl of Lipofectamine 2000 (Life Technologies) in 12.5 μl OptiMEM. The transfection mixture was incubated for 20 min in a 96-well plate before 1x10^5^ 293T in 75 μl DMEM was added to the well. At 48 h post-transfection, GFP fluorescent signal was measured. Subsequently, cells were lysed by Steady-Glo assay reagent (Promega) for 5 min before luminescence signal was measured. The polymerase activity was expressed as a ratio of luminescence to GFP signal.

### RNP reconstitution assays

RNP reconstitution assays were carried out as described previously [17]. HEK 293T cells (~10^6^ cells) were transfected with 1 μg each of plasmids encoding RNP subunits, including wild-type or mutant PB2s and full-length segment 6 (NA) vRNA templates or its truncated version (NA 47). Total RNA was extracted and analysed using ^32^P-labelled primers in reverse transcription [22]. Products were analysed by denaturing PAGE containing 7 M urea in Tris-borate-EDTA (TBE) buffer. Expression levels of PB2 and its mutants were checked by Western blotting with karyopherin ß3 as loading control.

### Circular dichroism spectroscopy of PB2 ‘627’ domain mutants

Purified wild-type and PB2 ‘627’ domain mutants were diluted to 0.3 mg/mL. Spectra were recorded at 25 ^o^C from 260 to 190 nm using a JASCO J-810 spectropolarimeter in a 0.1 cm path length cuvette. Data were collected every 0.2 nm, averaged over three scans and corrected for the baseline. Spectra were converted to molar ellipticity and plotted for comparison.

## Supporting information

S1 Appendix Datasets of figures. (XLSX)
